# Effects of 8-Week Orienteering Training on Physical Fitness Parameters among Adolescents Aged 14–18 Years

**DOI:** 10.1155/2022/5068599

**Published:** 2022-03-17

**Authors:** Özer Türkmen, Bilal Biçer

**Affiliations:** ^1^Antakya Yuksel Kemal Behzatoglu Anatolian High School, Turkey; ^2^School of Physical Education and Sport, Hatay Mustafa Kemal University, Turkey

## Abstract

The aim of this study is to examine the effects of 8-week orienteering training on physical fitness parameters in adolescents. To reveal this effect, this experimental study was designed as a pretest–posttest control group model. A total of 41 volunteers (20 females and 21 males) aged 14–18 years were divided into 2 groups: the orienteering training group (OTG) and control group (CG). Health-related and performance-related parameters of the physical fitness of the participants before and after the 8-week orienteering training were evaluated. After eight weeks of training, body weight (BW) increased by 1 kg on average in the OTG, but the body fat percentage (BFP) did not change. This increase could be due to the effect of the orienteering training. The CG, on the contrary, recorded an increase in BFP. Whereas both groups seemed similar in terms of elastic strength, and a significant improvement was found in the OTG in terms of anaerobic power, which considers BW. In terms of balance performance, the OTG showed a significant improvement, while the CG displayed a 97% rate of negative change. The positive increase rate in aerobic capacity was significantly higher in the OTG compared with the CG. Each participant in the OTG covered approximately 2000 meters engaging in parkour in each training session. Thus, the increase in aerobic capacity for the OTG can be explained by the number of 8-week training sessions. Rockport time decreased statistically in the OTG after training, but there was no difference in comparison with the CG. Moreover, similar results were observed in both the groups in body mass index, flexibility, agility, speed, and VO_2max_ values. Overall, orienteering training once a week for eight weeks resulted in positive developments in physical fitness parameters.

## 1. Introduction

Regular physical activity contributes positively to human health. However, with technology beginning to play an increasingly bigger role in daily life, individuals spend most of their time on computers, televisions, phones, tablets, video games, etc., which leads to a sedentary lifestyle without sufficient physical activity. Individuals who succumb to a sedentary lifestyle are faced with obesity, hypertension, diabetes, joint disorders, and cardiovascular and respiratory disorders. Further, psychological issues can also occur due to unhealthy food and unbalanced nutrition, stress, and anxiety. Those who indulge in regular physical activity are more energetic, able to perform daily activities without any problems, less prone to falling sick, and generally happier and healthier than those who do not. Physical fitness is defined as the ability to complete daily tasks comfortably and energetically without getting easily tired. Besides, it refers to having the energy to participate in leisure activities and respond to emergencies. Cardiovascular endurance, muscle strength, body composition, flexibility, speed, agility, coordination, and balance are considered components of physical fitness. These components can be improved through regular physical activity [1–3].

Children and adolescents spend most of their time in school, where they are physically and mentally more mobile and active than in the home environment. Accordingly, it can be concluded that schools are the most appropriate setting to teach students about physical fitness, how it can be improved, and the benefits of improving it. The general objective of physical fitness training is to provide students with the knowledge, skills, attitude, and behavior necessary for them to continue improving their physical fitness levels not only during school years but throughout their life, thereby minimizing the risk of health problems due to insufficient physical activity [4]. Physical activities for children and adolescents should not only be aimed at improving sports performance and success but also physiological and psychological health.

Orienteering, which has been gaining popularity day by day, is a physical activity suitable for all age groups that involves interaction between nature, sports, and humans. In physical fitness, mental strength plays an important role. Therefore, orienteering is also referred to as “running chess” [5]. Orienteering was initially regarded as an outdoor sport; however, with time, it has evolved into a favorable focus of nature, urban, and cultural tourism as a leisure-time activity. It has become a competitive sport, and orienteering championships are being organized. Orienteering can be performed outdoors, encompassing sportive performance parameters, such as speed, balance, reaction time, coordination, aerobic–anaerobic power, and strength, and it helps individuals' physical, mental, and social development [6]. These parameters improve quality of life, ensure healthy living, and increase the sports performance of individuals.

The increased usage of technology has resulted in a sedentary lifestyle for all age groups. The World Health Organization (WHO), along with many countries, is working to improve physical fitness levels of children and adolescents to prevent problems caused by a sedentary lifestyle. Activities such as orienteering that are performed in big outdoor spaces and different terrain types can be used to measure physical fitness levels more objectively. Although orienteering is a distinct sport as it is highly demanding in terms of cognitive and physical skills, it has received little attention in sports literature.

Upon examining recent literature on orienteering, it is seen that studies focus on factors such as problem-solving, visual reaction, and mental processes [7–9]. On the contrary, performance-related studies appear to be limited.

The present study was carried out to examine the effects of an 8-week orienteering training program on the physical fitness parameters among young people aged 14–18 years.

## 2. Materials and Methods

### 2.1. Participants

The number of participants in this study is 41, of which 20 are female and 21 are male high school students who participated voluntarily. While selecting participants for the research, the following criteria were considered: the participants should be healthy and not active in any sports. Necessary clarifications were made, and signed written consent was obtained. While outside the school, participants' parents managed their schedule (routine feeding habits, not participating in sports activities, etc.), and at school, teachers managed the program on not participating in physical activities. Participants were randomly divided into two groups, namely, the orienteering training group (OTG), consisting of nine females (16.22 ± 0.83 years) and eleven males (15.91 ± 1.22 years), and the control group (CG) consisting of ten females (16.80 ± 0.79 years) and eleven males (16.09 ± 1.04 years). All the participants completed the research process in full.

The participants in the OTG followed an orienteering training program, which included activities to improve orienteering knowledge and skills as well as relevant motor skills, once every weekend for 8 weeks; the sessions were 45–60 minutes long. The participants in the CG did not follow a training program. The main reason the orienteering training was done once a week was that the participants were preparing for their school exams and university entrance exams; they had limited free time. Therefore, participants' parents did not want their children to spend too much time on sports and physical activities. Yet, with planned and regular orienteering training once a week, the effects of such training on physical fitness parameters were measured.

### 2.2. Orienteering Training

The orienteering training sessions were conducted in a large campus-like space with slopes and smooth roads, a topographic map of which was drawn in the Antakya district of Hatay province. Throughout the track built in this area, 10 targets were placed randomly at approximately 100-meter intervals. Two points were designated to serve as the starting and finishing points. At the starting point, each participant was given a map showing the locations of the targets and was asked to reach the targets in a particular order as quickly as possible. The participants left the starting point at one-minute intervals. To check whether the participants completed the activity correctly, orienteering control punches were used on the map to mark the relevant points. At the finishing point, participants who had reached all 10 targets submitted their maps to the researcher. For each training exercise, different points on the map were marked as targets, and the participants were asked to decide on their own routes. The distance participants ran in their self-determined routes was measured. These activities were conducted with the OTG in a randomized manner each week at the specified location. The training sessions lasted between 45 and 60 minutes, including the warm-up and rest periods. Specifically, warm-up activities (hopping, jumping, arm swinging, etc.) took 8-10 minutes, stretching exercises (whole body) took 5-8 minutes, and the orienteering training session (reading the map and using the compass while walking, jogging, and running) took 40-45 minutes.

### 2.3. Procedures

This study adopted a pretest–posttest CG design. Health-related—body weight (BW), body fat percentage (BFP), body mass index (BMI), flexibility, elastic strength, VO_2max_, and aerobic capacity and performance-related—speed, agility, balance, coordination, and anaerobic power measurements were taken for participants before and after the 8-week orienteering training. Measurements were taken three days before the start of the study and three days after the end of the study. All tests were performed by expert researchers and assistants. Support was obtained from an expert female researcher to determine the skinfold thickness of female participants.

The BW (±0.1 kg) and height (±0.1 cm) of the participants were measured using a portable stadiometer (SECA, UK) while they were in standard sportswear and not wearing shoes. BMI (kg/m^2^) was calculated by dividing BW in kilograms by the square of height in meters. A manual skinfold caliper (Holtain, UK) was used to measure the BFP by determining skinfold thickness. Measurements were made from the chest, abdominal, and thigh areas of the male participants and the suprailiac, triceps, and thigh areas of the female participants. BFP was calculated using the body density and BFP formulas introduced by Jackson and Pollock [10].

The flexibility of the participants was determined by the sit-and-reach test. Participants were asked to touch the test bench with bare feet and wait for at least two seconds at the farthest point they could reach without bending their knees. The test was repeated twice and the best value was recorded in cm [11].

Elastic strength was measured with the counter movement jump using a jump meter tool (Seven, Turkey). The participants performed a maximal vertical jump on the jump mat and stood in the half-squat position after the jump without taking their hands off their waist. Participants were given two attempts after a trial jump, and the highest value was recorded in cm [12].

The VO_2max_ values of the participants were determined by performing the Rockport Gait Test. In this test, the participants were asked to walk on a one-mile-long (1,609 m) circular track as fast as they could. Participants' heart rate was monitored with a telemetric device. The finishing times were recorded as well. The participants' VO_2max_ values were calculated using the following formula, which included BW (lb), age (years), gendermale = 1, female = 0), one-mile-run completion time (minutes), and postexercise heart rate (beats/minute): estimated VO_2max_ (ml.kg^−1^.min^−1^) = 132.853 − 0.0769 × (Weight) − 0.3877 × (Age) + 6.315 × (Gender) − 3.2649 × (Time) − 0.1565 × (HR) [13].

The Yo-Yo Intermittent Recovery Test Level 1 was performed to determine the aerobic capacity of the participants. During this test, the participants were asked to perform 2 × 20 m runs, repeated at increasing speeds, with 10-second periods of active recovery [14]. The test started at the speed of 10 km/h and increased by 0.5 km/h at each level [15]. The tempo was monitored using an automatic acoustic device measuring the values at the start, during the return, and in the end. Aerobic capacity is defined as the maximum distance (m) traveled in cases where a participant fails twice to reach the finish line on time due to fatigue or interruption [14].

The speed levels of the participants were measured using the 30-meter sprint test, their agility levels using the Illinois agility test, their balance levels using the Flamingo Balance test, their coordination levels using the *t*-test, and their anaerobic capacity using the vertical jump test.

The elongated start method was adopted in the 30-meter sprint test. An electronic stopwatch (photocell) with a 0.01-second error margin was placed at the start and end points. All participants started the sprint from 50 cm behind the starting line when they felt ready. Each participant was given two attempts, and the best time was recorded in terms of seconds.

For the Illinois agility test, a 5-m wide and 10-m long track was prepared at the center of which four cones were placed at 3.3 m intervals. The test consisted of a 40-m straight and 20-m slalom run between the cones, with a 180° turn every 10 m. A two-door photocell electronic stopwatch with an error margin of 0.01 seconds was used to record the start and end times. In the test rounds, participants were given three attempts at a slow pace; then, the test was repeated twice and the best score was recorded in terms of seconds [16–19].

In the Flamingo Balance test, a metal beam 50 cm in length, 4 cm in height, and 3 cm in width was used. In this test, the participants were asked to stand barefoot on the beam for as long as possible in a flamingo-like pose. Which leg to stand on was decided by the participants. After starting, the timer was stopped each time the balance was disturbed and restarted when the balance was regained. Balance losses within a minute were counted, and if a participant lost balance more than 15 times in the first 30 seconds, the test was terminated and a score of 0 was given [20].

In the *t*-test, four funnels were arranged in a T shape. A distance of 4.57 m was left between the 3 funnels forming the upper line of the T shape. A funnel, which was set as the starting/finishing point and where a single-channel electronic stopwatch was placed, was placed at the bottom of the T shape, and the distance of this funnel to the funnel in the middle of the upper line was set to be 9.14 m. After the start command, the participants ran toward the funnel in the middle of the upper line and touched it and then continued by taking side steps toward the funnels placed on the sides and touched each funnel with their hands. The test was terminated by running back from the funnel in the middle of the upper line to the starting point [19, 21].

The anaerobic power of the participants was determined by using the values obtained from the vertical jump test in the Lewis formula (P = √4.9 × (Weight) × √D) [22].

### 2.4. Data Analysis

The IBM SPSS 23.0 (Statistical Package for Social Science) package software was used for the statistical analysis, and the arithmetic means, standard deviations, mean rank, and sum of ranks were calculated. The obtained data were tested for normality to use parametric statistical analysis. However, as the normality assumption did not meet the criteria to conduct two-way mixed model repeated measures ANOVA, we used the nonparametric equivalent tests. The Wilcoxon signed-rank test was performed for the intragroup comparison of participants' measurement results before and after the 8-week training, and the Mann–Whitney *U* test was performed to compare the pretest and posttest scores of the participants in the respective groups. As a result of these tests, the statistical significance level was found to be *p* < 0.05.

## 3. Results

Table 1 shows the changes in BW, BFP, and BMI after the 8-week orienteering training and the comparison of these parameters within and between groups.

*U*: Mann–Whitney *U*; ∗: *p* < 0.05; M: mean; SS: standard deviation; N/P/T: negative/positive/ties.

In the OTG, a statistically significant difference was found between the pretest and posttest results of the participants in terms of BW, whereas no changes were observed in terms of BFP after the 8-week period. On the contrary, although no change occurred in terms of the BW of the participants in the CG at the end of 8 weeks, a statistically significant increase of 3.81% was observed in the BFP (Table 1). Finally, no statistically significant difference was found in BMI values both within and between groups.

Participants in both groups showed improvement in terms of flexibility over the 8-week period. On the contrary, although no statistically significant difference was found between the groups, it is worth mentioning that the OTG participants' flexibility improved more than those in the CG. Participants in the OTG and CG were found to be at similar levels in terms of elastic strength.

Table 2 shows that the VO_2max_ values of the participants in both groups increased from the pretest to the posttest. Improvement was greater in the OTG (10.39%). A similar situation can be observed in aerobic capacity. Taking into consideration the distances covered in the Yo-Yo test, it was determined that the rate of improvement of the participants in the CG at the end of 8 weeks was 18.75%, whereas that of those in the OTG was 50.88% (*p* < 0.05).

Although there was no statistically significant difference between the groups in terms of the completion time of the Rockport test, the completion time of the participants in the CG did not change from the pretest to the posttest, whereas the completion time of those in the OTG decreased significantly (Table 2).

In both groups, a statistically significant change was observed in terms of speed and agility from the pretest to posttest (*p* < 0.05). When the speed and agility performances of the groups were compared, it was found that the participants in the OTG showed more improvement (Table 3).

The number of failed attempts of the participants in the OTG during the Flamingo Balance test decreased from the pretest to the posttest, whereas the contrary was observed in the CG with a 97% increase in the number of failed attempts. When the posttest results of the groups were compared, the number of failed attempts of the participants in the CG was found to be significantly higher than those in the OTG (*p* < 0.05).

The OTG participants' coordination time significantly reduced at the end of the 8-week training (*p* < 0.05). No significant change was observed in the CG in terms of coordination.

Anaerobic power levels of the participants in the OTG significantly increased from the pretest to the posttest. On the contrary, no statistically significant difference was observed in the anaerobic power levels of the participants in the CG after the 8-week period.

## 4. Discussion

It has been found that in the 8-week period, improvement was observed only in the OTG in terms of BW, Rockport test completion time, coordination, and anaerobic power, and deterioration was observed only in the CG in terms of BMI and balance. The groups were found to be similar in terms of BMI and elastic strength. However, although a significant change was observed within both groups from the pretest to posttest in terms of flexibility, VO_2 max_, speed, and agility, no significant difference was found between the groups. On the contrary, a significant improvement was observed in the OTG participants in terms of aerobic capacity, which was also significantly more than that of the CG.

Although there was no statistically significant difference between the groups with regard to many of the parameters at the end of the 8-week period, relatively more improvement was observed in the OTG in both health- and performance-related parameters of physical fitness. Although the fact that the orienteering training was conducted once a week can be regarded as a limitation, factors such as exam periods and parental restrictions offer the chance to make evaluations and generalizations under real-life conditions. Accordingly, it was found that orienteering training, even once a week, positively contributes to the physical fitness of adolescents, who may not have enough time to perform such activities frequently.

After the 8-week period, an increase was observed in the BWs of the participants in the OTG, while no change was seen in terms of BFP. In light of this finding, it is believed that the weight gain was caused by an increase in muscle mass. Although the BFP of the participants in the CG increased in a statistically significant manner, no significant difference was found between the groups. A similar situation was observed in terms of BMI values. The mean BMIs of the OTG and CG are (20.95 ± 3.21−20.88 ± 3.17 kg/m^2^) and (19.47 ± 2.62−19.24 ± 2.63 kg/m^2^), respectively, both of which fall under the “normal range” category according to the criteria accepted by the WHO. Upon examining the literature, it was seen that orienteering and soccer training practiced by participants in different age groups increased their BW from the pretest to the posttest [23–25]. An increase in BFP values was observed in studies conducted at different times and with different age groups with a focus on different sports (orienteering, skiing, football, and fitness), which was found to be higher in the CGs [26–28]. In different studies that were carried out during the weekly physical education class for 8 weeks [23], with late adolescent, young adult, and adult orienteering athletes [29] and with successful and unsuccessful orienteering athletes [26], no difference was found in terms of BMI values. All these studies support the finding of this study that as a result of orienteering training, BW increases significantly but no difference is noted in the BFP values. Thus, owing to orienteering training sessions that activate metabolic functions, fat is reduced and muscle mass is increased.

Flexibility is affected by factors such as age, psychological state (activity level and motivation), temperature, time of day, training and exercise level, muscle and neurophysiological characteristics (elasticity, muscle tone, and intramuscular and intermuscular coordination), fatigue, and warm-up level [30]. In light of the measurement results, it can be held that no differences were found between the groups because of differences in hormonal, neuromuscular, and physiological factors, as well as the fact that participants are in the period of adolescence and that no flexibility exercises were done in orienteering training sessions due to lack of time.

The participants of this study are high school students. The school attended by the participants is of a low–medium socioeconomic status. Students usually walk to school from their homes. Thus, students cover a certain distance on foot at a certain speed six days a week. This may be the reason the VO_2 max_ levels of the participants, who are at similar stages of development, were found to be comparable. It is possible that the improvement in the VO_2max_ levels of the participants in the OTG was more due to the increase in their muscle mass, and therefore, the BW. Studies carried out by Byars et al. [31] and Kim et al. [32] support the findings of our study.

Orienteering is an endurance-driven sport that can be performed in all kinds of terrains and differs from other running-based sports, especially depending on the type of terrain on which the sport is performed [6]. Due to the nature and rules of the sport, athletes determine their own routes by taking into account both the conditions of the terrain and the targets on their maps and race against time. Therefore, strong aerobic and anaerobic endurance is required to perform this sport [33]. Indeed, in a study, it was found that type I features are more common in the vastus lateralis and gastrocnemius mediale muscles of elite orienteering athletes [34]. Relevant studies revealed that the oxygen cost of running in the forest is approximately 25% higher compared to the road and that biomechanical differences, especially the stepping pattern, contribute to this increase [35–37]. In the track prepared for this study, which consists of a mixture of flat and rugged terrains, each participant covered an average distance of 2000 meters in each training session, which lasted 45–60 minutes. Therefore, the aerobic capacities of the participants in the OTG increased by an average of 230 m from the pretest to the posttest after the 8-week training. In support of these findings, there are studies in the literature showing that 6-week narrow pitch training [38] or, similarly, 5-week high intensity interval training or narrow-field game programs helped achieve better Yo-Yo test results [39].

Improvement was observed in terms of speed and agility in both groups, with participants in the OTG improving relatively more than those in the CG, though not in a statistically significant manner. This may be due to the muscle mass increase in the participants in OTG. Dündar defined speed as the physical value of the movement against external resistance that starts with a stimulus and is measured by the duration until the completion of the said movement [40]. In early adolescence and middle adolescence (14–18 years), depending on the mobility of nerve processes, the running speed of an individual reaches its full potential [41]. Agility is a rapid whole-body movement with a change in speed or direction in response to a stimulus [42]. Theoretically, the amount of body fat and the length of body segments can affect the level of agility. Of two athletes with the same BW, the one with higher BFP and less muscle mass needs to produce more force per unit of muscle during deflection and negative and positive acceleration due to high inertia [16, 42].

In terms of balance, a statistically significant change was found to have occurred in the CG. Further, a statistically significant difference was found between the groups in terms of the posttest results. The statistically significant difference between the groups was due to the fact that the participants in the OTG strengthened their balance skills throughout the 8-week training program, whereas the participants in the CG deteriorated in this sense in the same period. It is likely that the 8-week training program in which exercises including track running, jumping over hurdles and bumps, careful movement, and holding a specific pose for balance were done, and the additional muscle mass gained by the participants helped improve their balance skills. It was concluded in similar studies in the literature that such training programs help improve balance [26, 29, 43, 44]. It was found in similar studies carried out with participants from different age groups by performing other sports that balance can be improved with exercise and training [45–49].

From the pretest to the posttest, an improvement was observed in the OTG in terms of time needed for coordination, whereas no change was seen in the CG in that regard. Although a 4.3% improvement was observed within the OTG after the 8-week period, no significant difference was found between the groups. As limbs grow rapidly during puberty, individuals become less capable of using their hands and legs, which leads to the deterioration of their coordination skills [50]. Factors such as height, weight, balance, speed, age, and level of physical condition affect coordination. The fact that the ages of the participants vary between 14 and 18 explains the findings of this study on coordination. Indeed, in the studies of Çınar-Medeni et al. [29] conducted with late adolescent, young adult and adult orienteering athletes and of Padrón-Cabo et al. [51] conducted with adolescent football players in which the effects of coordination exercises performed using an agility ladder on physical fitness and technical performance were investigated, no significant difference was found between the OTG and CG in terms of coordination levels.

In the Lewis formula, which is used to measure the anaerobic power of athletes, both the jump height and BW of the individuals are taken into account. Table 1 shows that the mean bodyweight of the participants in the OTG is higher than that of those in the CG; however, the jump heights were found to be similar (Table 2). The participants in the OTG were able to improve their jump heights although their bodyweights increased, which points to increased anaerobic power. Despite the fact that no change was observed in the CG in this regard, no significant difference was found between the groups. These findings indicate how effective regular physical activity can be. The findings of this study are consistent with similar studies in the literature in terms of the effectiveness of training programs in this age group and similar age groups, as well as of detecting no significant difference between the OTG and CG [52, 53].

## 5. Conclusions

The participants in the OTG participated in the training sessions regularly, willingly, and happily, and at the end of the study, they gave positive feedback on how this training contributed to their life in terms of socialization and school success. After the study was completed, the participants continued with orienteering training. They participated in interschool orienteering competitions and ranked first and second in the province in three of the four categories.

We should also mention that the orienteering training took 8 weeks and was limited to adolescents. Similar studies with different age groups should be conducted to check if similar findings would be observed.

In conclusion, in a busy schedule comprising school, homework, and private lessons, accommodating an hour of orienteering training in a natural setting once a week will positively contribute to the physical fitness of adolescents.

## Figures and Tables

**Table 1:** Comparison of the BW (kg), BFP (%), and BMI (kg/m^2^) values within and between groups.

		Pretest M ± SS	Posttest M ± SS	Mean rank N/P/T	Sum of ranks N/P/T	*z*	*p*
BW	OTG	59.44 ± 11.79	60.59 ± 12.20	7.88/11.16/-	31.50/178.50/-	−2.745	0.006∗
	CG	56.13 ± 13.28	56.65 ± 13.20	9.71/11.64/-	68.00/163.00/-	−1.652	0.099
	*U*	175.00	163.50				
	*p*	0.361	0.225				
BFP	OTG	18.66 ± 9.15	18.75 ± 9.08	10.00/11.11/-	110.00/100.00/-	−0.187	0.852
	CG	15.20 ± 7.26	15.78 ± 7.21	8.33/12.07/-	50.00/181.00/-	−2.277	0.023∗
	*U*	160.00	163.00				
	*p*	0.192	0.220				
BMI	OTG	20.95 ± 3.21	20.88 ± 3.17	12.10/8.90/-	121.00/89.00/-	−0.597	0.550
	CG	19.47 ± 2.62	19.24 ± 2.63	12.92/8.44/-	155.00/76.00/-	−1.373	0.170
	*U*	166.00	151.00				
	*p*	0.251	0.124				

**Table 2:** Comparison of health-related parameters of physical fitness within and between groups.

		Pretest M ± SS	Posttest M ± SS	Mean rank N/P/T	Sum of ranks N/P/T	*z*	*p*
Flexibility (cm)	OTG	27.10 ± 7.45	33.65 ± 7.63	0.00/10.50/-	0.00/210.00/-	−3.932	0.001∗
	CG	23.33 ± 8.33	29.29 ± 11.40	10.00/11.11/-	20.00/211.00/-	−3.333	0.001∗
	*U*	162.50	164.00				
	*p*	0.215	0.230				
Elastic strength (cm)	OTG	27.00 ± 6.02	27.01 ± 6.06	9.22/9.78/2	83.00/88.00/2	−0.109	0.913
	CG	27.46 ± 5.85	27.29 ± 5.93	10.50/11.67/-	126.00/105.00/-	−0.365	0.715
	*U*	199.50	200.00				
	*p*	0.784	0.794				
VO_2max_ Ml/kg/min	OTG	52.05 ± 10.04	57.46 ± 6.76	1.00/10.50/1	1.00/189.00/1	−3.783	0.001∗
	CG	54.06 ± 7.61	57.62 ± 8.34	3.75/12.71/-	15.00/216.00/-	−3.493	0.001∗
	*U*	192.00	439.00				
	*p*	0.639	0.958				
Rockport time (min)	OTG	14.07 ± 1.90	13.28 ± 1.02	10.25/6.88/1	143.50/27.50/1	−2.527	0.012∗
	CG	13.41 ± 0.93	13.16 ± 1.14	11.31/9.00/1	147.00/63.00/1	−1.568	0.117
	*U*	183.00	189.50				
	*p*	0.481	0.786				
Aerobic capacity (m)	OTG	452.00 ± 146.88	682.00 ± 340.52	2.50/9.41/3	2.50/150.50/3	−3.513	0.001∗
	CG	396.19 ± 133.81	470.48 ± 192.63	4.38/9.88/5	17.50/188.50/5	−2.629	0.009∗
	*U*	159.50	131.50				
	*p*	0.186	0.040∗				

*U*: Mann–Whitney *U*; ∗: *p* < 0.05; M: mean; SS: standard deviation; N/P/T: negative/positive/ties.

**Table 3:** Comparison of performance-related parameters of physical fitness within and between groups.

		Pretest M ± SS	Posttest M ± SS	Mean rank N/P/T	Sum of ranks N/P/T	*z*	*p*
Speed (sec)	OTG	5.46 ± 0.63	5.20 ± 0.65	10.53/5.50/1	179.00/11.00/1	−3.382	0.001∗
	CG	5.40 ± 0.59	5.26 ± 0.61	10.59/10.13/1	169.50/40.50/1	−2.408	0.016∗
	*U*	199.50	197.00				
	*p*	0.784	0.735				
Agility (sec)	OTG	20.17 ± 1.80	19.10 ± 1.61	10.89/3.00/-	207.00/3.00/-	−3.808	0.001∗
	CG	19.76 ± 1.68	19.11 ± 1.36	13.13/5.67/-	197.00/34.00/-	−2.833	0.005∗
	*U*	174.500	205.500				
	*p*	0.354	0.907				
Balance (number of failed)	OTG	4.60 ± 6.08	3.85 ± 4.55	9.08/5.21/7	54.50/36.50/7	−0.630	0.528
	CG	3.95 ± 5.81	7.81 ± 5.17	7.80/10.15/3	39.00/132.00/3	−2.029	0.042∗
	*U*	195.000	116.000				
	*p*	0.065	0.012∗				
Coordination (sec)	OTG	13.53 ± 1.30	12.97 ± 1.19	10.75/8.25/-	193.50/16.50/-	−3.304	0.001∗
	CG	13.34 ± 1.20	13.26 ± 1.34	9.54/12.29/1	124.00/86.00/1	−0.709	0.478
	*U*	194.500	197.000				
	*p*	0.686	0.735				
Anaerobic power (kgm/sn)	OTG	132.08 ± 26.10	134.64 ± 27.00	8.00/11.13/-	32.00/178.00/-	−2.725	0.006∗
	CG	124.77 ± 29.42	125.91 ± 29.24	9.86/11.57/-	69.00/162.00/-	−1.616	0.106
	*U*	175.000	164.000				
	*p*	0.361	0.230				

*U*: Mann–Whitney *U*; ∗: *p* < 0.05; M:mean; SS: standard deviation; N/P/T: negative/positive/ties.

## Data Availability

The datasets analysed during the current study are available from the corresponding author on reasonable request.
